# Bayesian Estimation of Diagnostic Accuracy of Three Diagnostic Tests for Bovine Tuberculosis in Egyptian Dairy Cattle Using Latent Class Models

**DOI:** 10.3390/vetsci8110246

**Published:** 2021-10-21

**Authors:** Ibrahim Elsohaby, Jawher I. Alahadeb, Yasser S. Mahmmod, Marshal M. Mweu, Heba A. Ahmed, Mohamed M. El-Diasty, Attia A. Elgedawy, Eman Mahrous, Fatma I. El Hofy

**Affiliations:** 1 Department of Animal Medicine, Division of Infectious Diseases, Faculty of Veterinary Medicine, Zagazig University, Zagazig City 44511, Egypt; yasserpcr@gmail.com; 2 Department of Health Management, Atlantic Veterinary College, University of Prince Edward Island, Charlottetown, PE C1A4P3, Canada; 3 Department of Biology, College of Education (Majmaah), Majmaah University, P.O. Box 66, Al Majmaah 11952, Saudi Arabia; j.elhedeb@mu.edu.sa; 4 Department of Veterinary Sciences, Faculty of Health Sciences, Higher Colleges of Technology, Al Ain 17155, United Arab Emirates; 5 School of Public Health, College of Health Sciences, University of Nairobi, Nairobi 30197-00100, Kenya; marshal@uonbi.ac.ke; 6 Department of Zoonoses, Faculty of Veterinary Medicine, Zagazig University, Zagazig City 44511, Egypt; heba_ahmed@zu.edu.eg; 7 Mansoura Provincial Lab, Animal Health Research Institute, Mansoura 35516, Egypt; dr_mesbah_m@yahoo.com (M.M.E.-D.); dr.attia31@yahoo.com (A.A.E.); 8 Animal Health Research Institute, Giza 12618, Egypt; eman_mahrous12@yahoo.com; 9 Department of Bacteriology, Immunology and Mycology, Faculty of Veterinary Medicine, Benha University, Benha 13511, Egypt; fatma.abdlallah@fvtm.bu.edu.eg

**Keywords:** *Mycobacterium bovis*, test accuracy, dairy cows, Bayesian modelling, real-time PCR, rapid lateral flow

## Abstract

The aim of the present study was to calculate the sensitivity (Se) and specificity (Sp) of the single cervical tuberculin test (SCT), rapid lateral flow test (RLFT), and real-time polymerase chain reaction (RT-PCR) for the diagnosis of *Mycobacterium bovis* (*M. bovis*) infection in Egyptian dairy cattle herds within a Bayesian framework. The true *M. bovis* infection within-herd prevalence was assessed as a secondary objective. Data on the test results of SCT, RLFT, and RT-PCR for the detection of *M. bovis* were available from 245 cows in eleven herds in six major governorates in Egypt. A Bayesian latent class model was built for the estimation of the characteristics of the three tests. Our findings showed that Se of SCT (0.93 (95% Posterior credible interval (PCI): 0.89–0.93)) was higher than that of RT-PCR (0.83 (95% PCI: 0.28–0.93)) but was similar to the Se of RLFT (0.93 (95% PCI: 0.31–0.99)). On the contrary, SCT showed the lowest Sp estimate (0.60 (95% PCI: 0.59–0.65)), whereas Sp estimates of RT-PCR (0.99 (95% PCI: 0.95–1.00)) and RLFT (0.99 (95% PCI: 0.95–1.00)) were comparable. The true prevalence of *M. bovis* ranged between 0.07 and 0.71. In conclusion, overall, RT-PCR and RLFT registered superior performance to SCT, making them good candidates for routine use in the Egyptian bovine tuberculosis control program.

## 1. Introduction

Bovine tuberculosis (bTB) is an important zoonotic disease transmitted either directly via contact with infected animals or indirectly via the ingestion of contaminated raw or undercooked milk, milk products, meat, and meat products [1]. Bovine tuberculosis is a tuberculosis infection in cattle for which the primary causative agent is *Mycobacterium bovis* (*M. bovis*), which is a member of the *Mycobacterium tuberculosis complex* (MTC) [2]. Economic losses due to bTB in terms of the impact on productivity are notoriously difficult to assess. However, some studies have reported significant economic losses due to a reduction in milk production, weight, infertility, and meat condemnation as well as losses from mortality [3].

There are several methods for the diagnosis of bTB. These include direct techniques (detecting *M. bovis*), such as culture and PCR, or indirect methods that measure delayed hypersensitivity reactions, e.g., the skin test or the gamma interferon test [4]. Moreover, indirect methods may encompass an antibody response assessment through the application of serological tests such as ELISA and lateral flow immunoassays [5]. Lateral flow is a rapid, simple, and inexpensive assay. Consequently, it readily lends itself to use in low-income settings for the early detection of bTB [6]. Studies suggest that a combination of antigens may increase the test sensitivity (Se) without compromising its specificity (Sp) [7].

Despite the wide availability of tests for the identification of *M. bovis* infection at herd level, the diagnosis of bTB is difficult often because of the scarce diagnostic tests that fulfill all the essential criteria necessary for the identification of infected animals [8]. Moreover, notably, 20% of the new bTB cases are firstly diagnosed during post-mortem inspection at the slaughterhouse in cattle intended for human consumption [9]. Thus, the assessment of test diagnostic accuracy (Se and Sp) using classical evaluation methods is challenging. Mycobacterial culture has been applied as a reference test for evaluating the Se and Sp of other assays [10,11]. However, this conventional evaluation approach is associated with bias in test estimates due to the inherent imperfection of culture Se and Sp, which result in misclassification of the true infection status [10,11]. Bayesian latent class models (BLCMs) allow the quantification of diagnostic accuracy of tests in the absence of a perfect reference test [12,13]. Presently, the BLCMs have been accepted as a method of validation for animal infectious diseases diagnostics, including bTB diagnostics in the OIE standards [14].

In Egypt, the prevalence of bTB in large bovine ruminants, cattle, and buffaloes, ranged between 6.9% and 26.2% in the 1980s but declined to 2.6% in the 1990s. However, the prevalence of bTB has been rising lately due to the importation of live animals from endemic countries where the burden of *M. bovis* is high [15]. The Egyptian General Organization of Veterinary Services (GOVs) implements the single cervical tuberculin test (SCT) and slaughterhouse surveillance by visual inspection for the routine detection of new *M. bovis* infection in live and slaughtered animals, respectively [15]. The SCT has some practical shortcomings, including the subjectivity of the reading and interpretation of the test results and poor Sp [16,17,18]. Therefore, there is a need to implement more rapid and accurate diagnostic tests in the Egyptian bTB eradication program.

The primary objective of this study is to calculate the Se and Sp of SCT, rapid lateral flow test (RLFT), and real-time PCR (RT-PCR) for diagnoses of bTB in Egyptian dairy cattle herds using BLCMs. Secondarily, we aimed to estimate the true prevalences of *M. bovis* infection within the 11 herds involved in the present study.

## 2. Materials and Methods

### 2.1. Study Population

**Figure 1:**
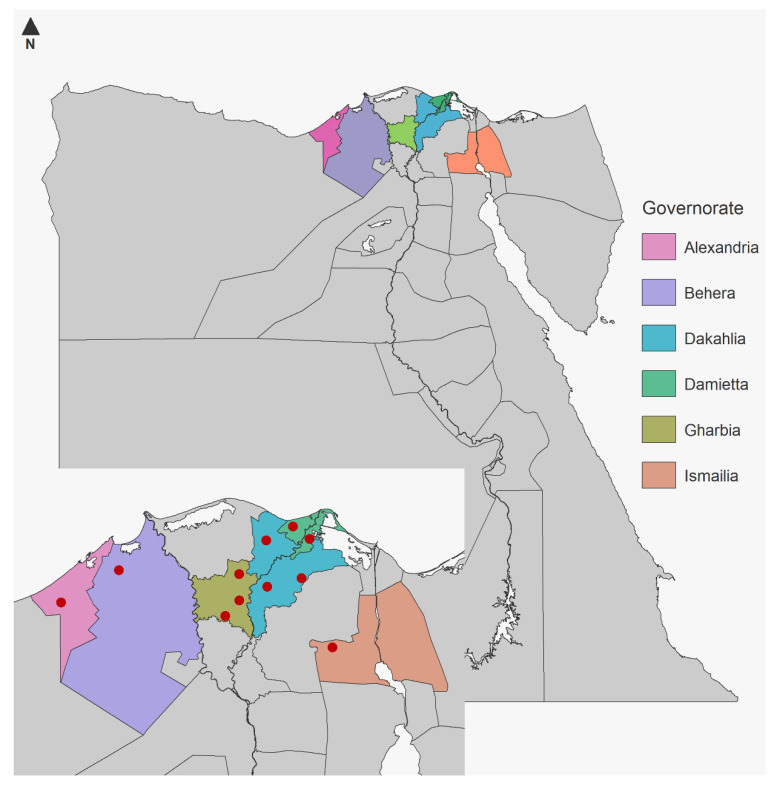
A map of Egypt and red dotes show the locations of herds from which samples were collected.

Eleven herds with Holstein dairy cows were selected for this study from a large routine national brucellosis and bTB surveillance program. Herds that had cows tested by SCT, RLFT, and RT-PCR were eligible for the study. All 2710 Holstein dairy cows in the selected eleven herds were tested by SCT. However, blood samples for RLFT and RT-PCR were collected only from 245 cows (including 215 SCT positive cows and 30 SCT negative cows). The 11 herds were distributed in six different Egyptian governorates, including two governorates in the West Nile Delta (2 herds, 131 cows (53.5%)) and four governorates in the East Nile Delta (9 herds, 114 cows (46.5%)) (Figure 1).

### 2.2. Sample Collection

During a cross-sectional study in 2017, blood samples (*n* = 245) were collected from selected dairy cows [19]. A sterile plastic tube containing Heparin was used to collect blood samples via tail vein puncture. The collected samples were labelled with basic information, such as cow ID, farm ID, and collection date. Samples were transported to the laboratory at room temperature (22 ± 3 °C) for later analysis. Dairy herd owners gave written informed consent to take part in the study. Available data were combined in a single database for this study, and cows with available findings for the SCT, RLFT, and RT-PCR were included.

### 2.3. Diagnostic Tests

#### 2.3.1. Single Cervical Tuberculin Test (SCT)

The SCT was carried out in accordance with the World Organization for Animal Health manual of standards [20]. Briefly, each cow received an intradermal injection of 0.1 mL mammalian purified protein derivative (PPD) tuberculin (VSVRI, Cairo, Egypt). After 72 h, the skin-fold thickness was measured using a calliper. Cows were classified SCT-positive if the skin-fold thickness at the injection site was >4 mm, negative if it <3 mm, and suspicious if it is between 3 and 4 mm [21].

#### 2.3.2. Rapid Lateral-Flow Test (RLFT)

To minimize false positive reactions, blood samples were taken from cows after SCT by 45 days. Samples were then examined by the RLFT (Anigen Bovine Tb Ab, BioNote Inc., Hwaseong-si, Korea) following manufacture instructions. The RLFT was utilized to identify anti-bovine tuberculosis (TB) antibodies (Ab) against the *M. bovis* mpb70 antigen. To perform the test, a ready-to-use disposable test kit was filled with 10 L of plasma and 3 drops of buffer (provided in the kit). The findings were read visually after 20 min. In the absence of a test band, the sample was declared negative (antibody-negative). However, if a test band other than the control line appeared in the test area, the sample was then deemed positive (antibody-positive).

#### 2.3.3. Real-Time PCR (RT-PCR)

The QIAamp DNA Mini kit (Qiagen, Hilden, Germany) was used to extract DNA from blood samples, which was then submitted to RT-PCR using the MTplex dtec-RT-qPCR Test (Edifici-Quórum3, Elche, Spain) according to the manufacturer’s instructions. Reaction mixtures with no additional DNA were conducted in the same reaction as negative controls. Another reaction mixture was conducted, this time with an undiluted positive control (Standard Template MTplex positive control) and a duplicate of two-fold dilutions of the positive control. In an Applied Biosystem StepOne RT-PCR System, the PCR reactions were carried out under the following optimal cycle conditions: 95 °C for 5 min followed by two steps 45 cycles of 95 °C for 30 s and 60 °C for 60 s for hybridization, extension and data collection. StepOneTM software version 2.2.2 was used to gather the FAM fluorogenic signal and to determine the cycle threshold of the reactions (Life Technology, Warrington, UK). To consider the sample positive, three distinctive phases (geometric, linear and plateau) had to be shown in the corresponding amplification curve, which characterized the progression of the PCR reaction.

### 2.4. Post-Mortem Examination

All RLFT reactor cows (*n* = 65) were slaughtered. Official veterinarians inspected slaughtered cows for bTB post-mortem lesions following the abattoir’s standard operating procedures.

### 2.5. Statistical Analysis

According to the guidelines for reporting diagnostic accuracy studies (STARD-BLCMs) [13], a BLCM fitted in OpenBUGS v3.2.2 [22] was implemented to evaluate Se and Sp estimates of the three tests (SCT, RLFT, and RT-PCR), in addition to the true prevalence of *M. bovis* infection in 11 cattle populations. The model was implemented in R software v3.4.3 using the ‘BRugs’ package v0.9-0 [22] and is included in Supplementary File S1.

Three assumptions are essential while constructing BLCMs [23]. The target population should first be divided into two or more subpopulations, each with a different prevalence. The 11 dairy herds in this study were shown to have variable prevalences (perhaps due to differences in farm management practices [24]), resulting in distinct subpopulations. Secondly, the tests Se and Sp should be consistent across subpopulations. To test this hypothesis, particular subpopulations were systematically excluded from the models, and the test’s Se and Sp were re-estimated. Lastly, the diagnostic tests are believed to be conditionally independent based on the disease status. This was considered biologically true in our case, as the three tests use distinct detection mechanisms. However, we tested for dependence by adding two conditional covariance parameters, σ_se_ and σ_sp_, between each pair of the Se and Sp of the tests, respectively, as specified by Gardner et al. [25]. The covariances were tested with a Bayesian p-value for deviations from zero (zero covariance representing conditional independence). Additionally, the relative fitness of the models (assuming independence vs. dependence) were compared using a deviance information criterion (DIC), with the model recording a lower DIC being preferred.

Counts (*O_k_*) of the several test combinations (for example +, +, +) were assumed to have a multinomial distribution:

(1) $$O_{k}|{\mathrm{Se}}_{q}{\mathrm{Sp}}_{q}P_{k}\;\sim \;multinomial\left(prob_{k},n_{k}\right)\;$$

where ${\mathrm{Se}}_{q}$ and ${\mathrm{Sp}}_{q}$ reflect the respective test characteristics q (q=1:3) in subpopulation k and $P_{k}$ is the true prevalence estimate for the kth (k=1:11) subpopulation. $Prob_{k}$ is a vector of probabilities of observing the different combinations of test results, and $n_{k}$ reflects the number of cows tested for the kth subpopulation.

The 11 subpopulations gave 77 degrees of freedom, which was sufficient to estimate 17 parameters (Se and Sp of the three tests, as well as 11 subpopulation prevalences), showing that the model was identifiable. Notably, if the number of subpopulations (k) and tests (q) satisfy the equation: $k\geq q/\left(2^{q-1}-1\right)$, the identifiability argument can be supported [26].

Since, to the best of our knowledge, no available literature exists on the diagnostic Se and Sp estimates for *M. bovis* infection from similar farm management conditions in developing countries, vague priors for Se $\left[{\mathrm{Se}}_{1}\;\sim \;uniform\left(0.560,\;0.933\right)\right]$ and Sp $\left[{\mathrm{Sp}}_{1}\;\sim \;uniform\left(0.593,\;1.000\right)\right]$ of SCT (test 1) were specified based on diagnostic information from developed settings [10,27,28].

For each test, the Youden’s index (Y) was produced to compare the overall performance of the diagnostic tests. The test shows the highest value is generally regarded as the most recommended [29]:

(2) $$Y_{q}=\left({\mathrm{Se}}_{q}+{\mathrm{Sp}}_{q}\right)-1$$

Two Markov Chain Monte Carlo chains (MCMC) with different values were used to initialize the model. A total of 80,000 samples were used in each chain, with the first 30,000 being eliminated as burn-in. Visual inspection of Gelman–Rubin diagnostic plots, density plots, time series plots of selected variables, and autocorrelation plots were used to test MCMC chain convergence using two-sample chains with varying initial values. [30]. The goodness-of-fit of the Bayesian model was evaluated using the posterior predictive *p*-value–with values between 0.05 and 0.95 suggesting acceptable fit [31]. The median and associated 95% posterior credible intervals (PCI) were used to report the Se and Sp of the tests, as well as the posterior distribution of the true prevalences.

## 3. Results

### 3.1. Descriptive Results

In total, blood samples were collected from 245 cows in 11 dairy herds in Egypt. The SCT, RLFT, and RT-PCR results were available for the 245 cows involved in the study. However, the post-mortem results were available for the 65 RLFT positive cows. The distribution of the results for bTB ante- and post-mortem tests are presented in Table 1.

### 3.2. Bayesian Models

The results of cross-tabulated (contingency) counts of the dichotomous of SCT, RLFT, and RT-PCR for the *M. bovis* detection are listed in Table 2. Table 3 shows the Se and Sp estimates of the three tests, the posterior median, and 95% PCI of true prevalence. The tests characteristics and the prevalences within the herds were obtained using the uninformative prior models. Of note, the model assuming conditional independence was preferred based on its lower DIC value.

Se of SCT (0.93 (95% PCI: 0.89–0.93)) was higher than Se of RT-PCR (0.83 (95% PCI: 0.28–0.93)) but it was comparable to the Se of RLFT (0.93 (95% PCI: 0.31–0.99)). On the contrary, SCT showed the lowest Sp estimate (0.60 (95% PCI: 0.59–0.65)), whereas the Sp estimates of RT-PCR (0.99 (95% PCI: 0.95–1.00)), and RLFT (0.99 (95% PCI: 0.96–1.00)) were comparable. The true within-herd prevalence estimates of *M. bovis* infection ranged from 0.07 (95% PCI: 0.003–0.51) in Herd10 to 0.71 (95% PCI: 0.47–0.94) in Herd2 (Table 3). Based on overall test performances, the RLFT performed similarly to RT-PCR (Y. index: 0.92 vs. 0.82), but better than SCT (Y.index: 0.52). Notably, the tests were found to be conditionally independent. The Bayesian model fitted the data well based on the posterior predictive values, except for diagnostic data from herds 4, 6, and 10.

### 3.3. Post-Mortem Results

Out of the 65 slaughtered cows, 49 exhibited visible lesions in lymph nodes and internal organs, including enlargement and focal to multifocal yellow to grey casiated cheese-like nodules. There were no visible lesions on the remaining 16 cows.

## 4. Discussion

The assessment of accuracy diagnostic tests for the diagnosis of bTB is challenging due to the absence of perfect gold standards for live-animal testing. Many diagnostic tests, including culture, histopathology, PCR, and even SCT, have been used as a reference test (pseudo-gold standards) for classical validation of the tests used for the detection of *M. bovis* infection in cattle [19,32,33]. However, these methods are associated with many reported biases [30,34]. The cornerstone of preventing bTB is the price of detection and eradication of the infected animals [35]. Thus, BLCMs have been used to assess the performance of diagnostic tests for the detection of *M. bovis* infection in dairy cows with unknown true infection status.

Estimates of Se and Sp of SCT, RLFT, and RT-PCR for the diagnosis of bTB in the blood of dairy cows under intensive system production in Egypt were estimated. Cows were tested first with SCT. Then, blood samples were collected after 45 days for RLFT and RT-PCR testing. This sampling approach aligns with previous studies [36,37,38], which reported that the diagnostic accuracy of the serological test was reduced if the test was performed prior to the tuberculin test. In the Spanish study, blood samples for serological testing were collected prior to and 15 days post-tuberculin test and the Se estimates were higher for post- (66.7–85.2%) than prior- (23.9–32.6%) tuberculin test [36,37]. These results suggest that blood sampling after tuberculin testing maximizes the beneficial effects of serological testing.

In the current study, Se estimates of SCT were relatively similar to 95.2% Se reported in dairy cows in Thailand [39], and higher than the 88.6% [28] and 57.7% [27] Se reported in Northern Ireland, the 80.3% Se reported in some French departments [40], and the 60.8% Se reported in Irish dairy farms [41]. However, it should be noted that the high Se estimate of SCT reported here could be due to the features of the test population. The cows included in this study are derived from chronically infected herds that have been infected for a long time or have been infected multiple times. The SCT in the present study showed the highest Se (93%), similar to the RLFT (93%). Similar results have been reported in bison [42,43]. Furthermore, Cousins and Florisson [44] reported that the skin test has an equal or higher Se estimate than the serological test. Although the low Se seems to be a common characteristic of serological tests in cattle [45] and other species [46], the high Se of RLFT (93%) may be attributed to cows that were chronically infected. The Se and Sp of RLFT reported in this study were higher than the 80.7% Se and 84.2% Sp reported in cattle in Turkey using an IFN-γ assay as reference test [7]. The high Sp of RLFT allows it to be used in the early detection of latent infected cattle [6]. In this study, the Se and Sp estimates of RT-PCR were relatively higher than that of the conventional PCR using either blood (Se = 68% and Sp = 98%) or milk (Se = 29% and Sp = 88%) samples from the same population [47]. Furthermore, the Se and Sp of RT-PCR were higher than 76.7% Se and similar to the 99.3% Sp reported in another study in Spain using tissue samples [48].

A high within-herd prevalence ranged from 7% to 71% was observed in our study, which is similar to the 68.8% reported in a recent study [49] and significantly greater than that reported in previously published studies [15,50,51]. The variation in bTB prevalence could be attributed to the management practices, breed, and geographical distribution of each study. Cattle importation, which is responsible for the emergence of new strains of *M. bovis* in Egypt, may have a role in the high frequency of bTB found in this study [50]. The animals used in this investigation were females, and the incidence of bTB infection among females was much higher than that of males, owing to the fact that females are typically bred for longer periods of time than males and therefore subjected to repeated infection [52]. Furthermore, the Egyptian bTB eradication program uses the SCT, which is affected by the presence of other infectious diseases such as Johnes disease [53] and the high number of cows in the late phase of the disease [54].

## 5. Conclusions

Without assuming the existence of a perfect reference standard, we estimated the Se and Sp of SCT, RLFT, and RT-PCR for *M. bovis* infection detection in Egyptian dairy cattle. RT-PCR and RLFT demonstrated better performance than SCT, making them good candidates for routine use in the control program of bTB in Egypt.

## Figures and Tables

**Table 1:** Distribution of results for three ante-mortem tests (single cervical tuberculin test (SCT), rapid lateral-flow test (RLFT) and Real-Time PCR (RT-PCR)) and post-mortem test for diagnosis of bovine tuberculosis in dairy cattle (*n* = 245) from 11 Egyptian cattle herds.

Tests	Number (%) of Test Positive	Number (%) of Test Negative	Total
SCT	215 (87.8)	30 (12.2)	245
RLFT	65 (26.5)	180 (73.5)	245
RT-PCR	59 (24.1)	186 (75.9)	245
Post-mortem	49 (75.4)	16 (24.6)	65

**Table 2:** Cross-tabulated findings for combinations of the three diagnostic tests (single cervical tuberculin test (SCT), rapid lateral-flow test (RLFT) and Real-Time PCR (RT-PCR)) for diagnosis of bovine tuberculosis in dairy cattle (*n* = 245) from 11 Egyptian cattle herds.

Herds	Tests Combinations (SCT T1; RLFT T2; RT-PCR T3)								Total
	+++	++−	+−+	+−−	−++	−+−	−−+	−−−	
Herd1	6	0	0	4	0	0	0	3	13
Herd2	12	1	0	3	0	0	0	3	19
Herd3	1	0	1	6	0	0	0	3	11
Herd4	0	0	0	5	0	0	0	3	8
Herd5	3	1	0	6	0	0	0	3	13
Herd6	0	0	1	9	0	0	0	3	13
Herd7	2	0	0	8	0	0	0	3	13
Herd8	5	0	0	5	0	0	0	3	13
Herd9	3	0	1	4	0	0	0	3	11
Herd10	0	0	0	8	0	0	0	3	11
Herd11	24	7	0	89	0	0	0	0	120
Total	56	9	3	147	0	0	0	30	245

(+) = test positive; (−) = test negative.

**Table 3:** Test estimates of three diagnostic tests (single cervical tuberculin test (SCT), rapid lateral-flow test (RLFT) and Real-Time PCR (RT-PCR)) for diagnosis of bovine tuberculosis in dairy cattle (*n* = 245) from 11 Egyptian cattle herds.

Parameters ^1^	Test Estimates (SCT T1; RLFT T2; RT-PCR T3)	
	Median	95% PCI ^2^
Se_SCT_	0.93	0.89–0.93
Se_RLFT_	0.93	0.31–0.99
Se_RT-PCR_	0.83	0.28–0.93
Sp_SCT_	0.60	0.59–0.65
Sp_RLFT_	0.99	0.96–1.00
Sp_RT-PCR_	0.99	0.95–1.00
Herd1	0.51	0.24–0.86
Herd2	0.71	0.47–0.94
Herd3	0.23	0.04–0.71
Herd4	0.09	0.003–0.49
Herd5	0.36	0.13–0.81
Herd6	0.11	0.004–0.64
Herd7	0.22	0.051–0.72
Herd8	0.44	0.19–0.83
Herd9	0.40	0.14–0.80
Herd10	0.07	0.003–0.51
Herd11	0.28	0.19–0.99
y.index[SCT]	0.52	0.48–0.58
y.index[RLFT]	0.92	0.28–0.99
y.index[RT-PCR]	0.82	0.25–0.92

^1^ Se = sensitivity; Sp = specificity; Herd1–Herd11: within-herd prevalence of bovine tuberculosis. ^2^ 95% Posterior credible interval (PCI).

## Data Availability

The data presented in this study are available on request from the corresponding author.

## Supplementary Materials

The following are available online at https://www.mdpi.com/article/10.3390/vetsci8110246/s1, File S1: Bayesian Estimation of Diagnostic Accuracy of Three Diagnostic Tests for Bovine Tuberculosis in Egyptian Dairy Cattle Using Latent Class Models.
