# Supplementary material for: Bayesian Estimation of Diagnostic Accuracy of Three Diagnostic Tests for Bovine Tuberculosis in Egyptian Dairy Cattle Using Latent Class Models
Source: Vet Sci. 2021 Oct 21;8(11):246. doi: 10.3390/vetsci8110246 (PMC8622144; doi:10.3390/vetsci8110246)
Supplement: Supplementary file 1 [file vetsci-08-00246-s001.zip › vetsci-1322236-supplementary.pdf]

Supplementary File S1

```
#BOVINE TB DIAGNOSTICS EVALUATION

rm(list=ls())

setwd("C:...........")

require('BRugs')

tb.dat <- read.csv("TB_data_4_Tests.csv",header=T)

str(tb.dat)

table(tb.dat$location) #See nos. of cows per herd

#####################################################################################
#######################

##################
#Model with priors
##################

model.tb.1 <- function(){

  #priors: #Test 1=Tuberculin; Test 2=Rapid test; Test 3=PCR

  se[1] ~ dunif(0.560,0.933) #uniform priors for the Tuberculin test
  sp[1] ~ dunif(0.593,1.000)
```

```
for(i in 2:3){

  se[i] ~ dbeta(1,1)
  sp[i] ~ dbeta(1,1)

}

for(i in 1:11){

  p[i] ~ dbeta(1,1)

  pop[i,1:8] ~ dmulti(par[i,1:8],n[i])
  pop.new[i,1:8] ~ dmulti(par[i,1:8],n[i]) #create new variable to monitor

  par[i,1] <- se[1]*se[2]*se[3]*p[i] + (1-sp[1])*(1-sp[2])*(1-sp[3])*(1-p[i])
  par[i,2] <- se[1]*se[2]*(1-se[3])*p[i] + (1-sp[1])*(1-sp[2])*sp[3]*(1-p[i])
  par[i,3] <- se[1]*(1-se[2])*se[3]*p[i] + (1-sp[1])*sp[2]*(1-sp[3])*(1-p[i])
  par[i,4] <- (1-se[1])*se[2]*se[3]*p[i] + sp[1]*(1-sp[2])*(1-sp[3])*(1-p[i])
  par[i,5] <- se[1]*(1-se[2])*(1-se[3])*p[i] + (1-sp[1])*sp[2]*sp[3]*(1-p[i])
  par[i,6] <- (1-se[1])*se[2]*(1-se[3])*p[i] + sp[1]*(1-sp[2])*sp[3]*(1-p[i])
  par[i,7] <- (1-se[1])*(1-se[2])*se[3]*p[i] + sp[1]*sp[2]*(1-sp[3])*(1-p[i])
  par[i,8] <- (1-se[1])*(1-se[2])*(1-se[3])*p[i] + sp[1]*sp[2]*sp[3]*(1-p[i])

  check[i] <- pop.new[i,1:8] - pop[i,1:8]
  post.pred.chk[i] <- step(sum(check[i])) #posterior predictive value: observed and simulated data
should be equal

  n[i] <- sum(pop[i,1:8])
```

```
  }

  for(i in 1:3){

    y.index[i] <- (se[i] + sp[i]) - 1 #Youden's index

  }

  p.se[1] <- step(se[1] - se[2]) #Tuberculin & Rapid test
  p.se[2] <- step(se[1] - se[3]) #Tuberculin & PCR
  p.se[3] <- step(se[2] - se[3]) #Rapid & PCR

  p.sp[1] <- step(sp[1] - sp[2])
  p.sp[2] <- step(sp[1] - sp[3])
  p.sp[3] <- step(sp[2] - sp[3])

}

######################
#Model with no priors
######################

model.tb.2 <- function(){

  #priors: #Test 1=Tuberculin; Test 2=Rapid test; Test 3=PCR

  for(i in 1:3){
```

```
  se[i] ~ dbeta(1,1)

  sp[i] ~ dbeta(1,1)

}

for(i in 1:11){

  p[i] ~ dbeta(1,1)

  pop[i,1:8] ~ dmulti(par[i,1:8],n[i])

  pop.new[i,1:8] ~ dmulti(par[i,1:8],n[i])

  par[i,1] <- se[1]*se[2]*se[3]*p[i] + (1-sp[1])*(1-sp[2])*(1-sp[3])*(1-p[i])

  par[i,2] <- se[1]*se[2]*(1-se[3])*p[i] + (1-sp[1])*(1-sp[2])*sp[3]*(1-p[i])

  par[i,3] <- se[1]*(1-se[2])*se[3]*p[i] + (1-sp[1])*sp[2]*(1-sp[3])*(1-p[i])

  par[i,4] <- (1-se[1])*se[2]*se[3]*p[i] + sp[1]*(1-sp[2])*(1-sp[3])*(1-p[i])

  par[i,5] <- se[1]*(1-se[2])*(1-se[3])*p[i] + (1-sp[1])*sp[2]*sp[3]*(1-p[i])

  par[i,6] <- (1-se[1])*se[2]*(1-se[3])*p[i] + sp[1]*(1-sp[2])*sp[3]*(1-p[i])

  par[i,7] <- (1-se[1])*(1-se[2])*se[3]*p[i] + sp[1]*sp[2]*(1-sp[3])*(1-p[i])

  par[i,8] <- (1-se[1])*(1-se[2])*(1-se[3])*p[i] + sp[1]*sp[2]*sp[3]*(1-p[i])

  check[i] <- pop.new[i,1:8] - pop[i,1:8]

  post.pred.chk[i] <- step(sum(check[i]))

  n[i] <- sum(pop[i,1:8])

}

for(i in 1:3){
```

```
    y.index[i] <- (se[i] + sp[i]) - 1 #Youden's index

  }

  p.se[1] <- step(se[1] - se[2]) #Tuberculin & Rapid test
  p.se[2] <- step(se[1] - se[3]) #Tuberculin & PCR
  p.se[3] <- step(se[2] - se[3]) #Rapid & PCR

  p.sp[1] <- step(sp[1] - sp[2])
  p.sp[2] <- step(sp[1] - sp[3])
  p.sp[3] <- step(sp[2] - sp[3])

}

############################################################################################################################################

#Data

data.tb <- matrix(NA,ncol=8,nrow=length(unique(tb.dat$farm)))

for(p in 1:length(unique(tb.dat$farm))){

  data.tb[p,1] <- nrow(tb.dat[tb.dat$tuberculin==1 & tb.dat$rapid_test==1 & tb.dat$RT_PCR==1 & tb.dat$farm==p,])
  data.tb[p,2] <- nrow(tb.dat[tb.dat$tuberculin==1 & tb.dat$rapid_test==1 & tb.dat$RT_PCR==0 & tb.dat$farm==p,])
  data.tb[p,3] <- nrow(tb.dat[tb.dat$tuberculin==1 & tb.dat$rapid_test==0 & tb.dat$RT_PCR==1 & tb.dat$farm==p,])
```

```
  data.tb[p,4] <- nrow(tb.dat[tb.dat$tuberculin==0 & tb.dat$rapid_test==1 & tb.dat$RT_PCR==1 &
tb.dat$farm==p,])

  data.tb[p,5] <- nrow(tb.dat[tb.dat$tuberculin==1 & tb.dat$rapid_test==0 & tb.dat$RT_PCR==0 &
tb.dat$farm==p,])

  data.tb[p,6] <- nrow(tb.dat[tb.dat$tuberculin==0 & tb.dat$rapid_test==1 & tb.dat$RT_PCR==0 &
tb.dat$farm==p,])

  data.tb[p,7] <- nrow(tb.dat[tb.dat$tuberculin==0 & tb.dat$rapid_test==0 & tb.dat$RT_PCR==1 &
tb.dat$farm==p,])

  data.tb[p,8] <- nrow(tb.dat[tb.dat$tuberculin==0 & tb.dat$rapid_test==0 & tb.dat$RT_PCR==0 &
tb.dat$farm==p,])


}


data.tb <- list(data.tb); names(data.tb) <- 'pop'


##########################################################################################
########################################


#Convert all to BUGS format


results.tb <- list(); models.x <- c(model.tb.1,model.tb.2); priors <- c("prior","no.prior"); dic.vals <- c()


for(m in 1:2){


  mod.tb <- assign(paste("model.tb.",m,sep=""),models.x[[m]])


  #write model to a file

  writeModel(mod.tb,'model.tb.txt')


  #Bugs data

  bugsData(data.tb,fileName='data.tb.txt')
```

```
#make 2 initial values chains

bugsInits(inits=list(list(se=rep(0.80,times=3),sp=rep(0.80,times=3),p=rep(0.15,times=11))),numChains=
1,'CID.Init1.txt')

bugsInits(inits=list(list(se=rep(0.90,times=3),sp=rep(0.90,times=3),p=rep(0.35,times=11))),numChains=
1,'CID.Init2.txt')

#now check, load data, compile etc.
modelCheck('model.tb.txt') #check model file.
modelData('data.tb.txt') #read data file
modelCompile(numChains=2) #compile model with 2 chains
modelInits('CID.Init1.txt',1) #read init data file
modelInits('CID.Init2.txt',2) #read init data file
modelGenInits() #generate the missing initial values

modelUpdate(30000) #burn in

samplesSet(c('se','sp','p','y.index','p.se','p.sp','post.pred.chk')) #parameters to monitor

modelUpdate(50000) #more iterations

#SOME DIAGNOSTICS FIRST
#Check convergence (Trace plots) - should ideally check all
#samplesHistory('se',mfrow=c(1,1)) # plot the chain,
#samplesHistory('sp',mfrow=c(1,1)) # plot the chain,
#samplesHistory('p',mfrow=c(1,1))

#Plot the Gelman-Rubin diagnostic statistics - ratio should be close to 1
```

```
  #samplesBgr('se',mfrow=c(1,1))

  #samplesBgr('sp',mfrow=c(1,1))

  #samplesBgr('p',mfrow=c(1,1))


  #Density plots

  #samplesDensity('se',mfrow=c(1,1))

  #samplesDensity('sp',mfrow=c(1,1))

  #samplesDensity('p',mfrow=c(1,1))


  results.tb[[m]] <- samplesStats('*'); names(results.tb)[m] <- priors[m]


  dicSet(); modelUpdate(50000) #monitor DIC and do further model updates


  dic.vals[m] <- dicStats()[1,3] #show DIC value (compares prior vs non.prior models)


}


capture.output(results.tb,file='all.TB.results.txt')


dic.vals #


##########################################################################################
####################################

#END
```
